# Lubiprostone as a potential therapeutic agent to improve intestinal permeability and prevent the development of atherosclerosis in apolipoprotein E-deficient mice

**DOI:** 10.1371/journal.pone.0218096

**Published:** 2019-06-17

**Authors:** Kentaro Arakawa, Tomoaki Ishigami, Michiko Nakai-Sugiyama, Lin Chen, Hiroshi Doi, Tabito Kino, Shintaro Minegishi, Sae Saigoh-Teranaka, Rie Sasaki-Nakashima, Kiyoshi Hibi, Kazuo Kimura, Kouichi Tamura

**Affiliations:** 1 Department of Medical Science and Cardiorenal Medicine, Yokohama City University, School of Medicine, Kanagawa, Japan; 2 Division of Cardiology, Yokohama City University Medical Center, Kanagawa, Japan; Max Delbruck Centrum fur Molekulare Medizin Berlin Buch, GERMANY

## Abstract

The interaction between atherosclerosis and commensal microbes through leaky gut syndrome (LGS), which is characterized by impaired intestinal permeability and the introduction of undesired pathogens into the body, has not been fully elucidated. Our aim was to investigate the potential role of a ClC-2 chloride channel activator, lubiprostone, which is reported to have beneficial effects on LGS, in the development of atherosclerosis in apolipoprotein E–deficient (ApoE-/-) mice. After a 15-week feeding period of a Western diet (WD), ApoE-/- mice were treated with a Western-type diet (WD) alone or WD with oral supplementation of lubiprostone for 10 weeks. This feeding protocol was followed by experimental evaluation of LGS and atherosclerotic lesions in the aorta. In mice with lubiprostone, *in vivo* translocation of orally administered 4-kDa FITC-dextran was significantly improved, and RNA expression of the epithelial tight junction proteins, Zo-1 and occludin, was significantly up-regulated in the ileum, compared to the WD alone group, suggesting a possible reversal of WD-induced intestinal barrier dysfunction. As a result, WD-induced exacerbation of atherosclerotic lesion formation was reduced by 69% in longitudinally opened aortas and 26% in aortic root regions. In addition, there was a significant decrease in circulating immunoglobulin level, followed by an attenuation of inflammatory responses in the perivascular adipose tissue, as evidenced by reduced expression of pro-inflammatory cytokines and chemokines. Lubiprostone attenuates atherosclerosis by ameliorating LGS-induced inflammation through the restoration of the intestinal barrier. These findings raise the possibility of targeting LGS for the treatment of atherosclerosis.

## Introduction

Atherosclerosis is the leading cause of cardiovascular mortality and morbidity worldwide. Recent studies have revealed that atherosclerosis arises from a systemic inflammatory process, including the accumulation and the activities of immune cells [1, 2]. In our previous work, we reported that atherosclerotic development, caused by intestinal microbiome, is due to the recruitment and ectopic activation of B2 cells in the perivascular adipose tissue and an increase in circulating immunoglobulin (IgG and IgG3) [3,4]. This inflammatory pathway can be inhibited by antibiotic-induced elimination of the intestinal microbiome, as well as by depletion of B2 cells with antibodies against the cell surface antigen CD23. These findings underline a possible persistent inflammatory process in atherosclerosis, centered on a pathological humoral immunity between commensal microbes and activated sub-populations of substantial B cells in the vicinity of the arterial adventitia.

The intestinal barrier is a multi-layer defense system, with the lumen providing the first line of defense, where enzymatic degradation of bacteria and antigens occurs and the commensal bacteria prevent colonization of pathogens [5]. Although this barrier acts continuously to avoid translocation of intestinal pathogens, this function may be altered by endogenous or exogenous factors, including lipopolysaccharide (LPS), alcohol consumption [6], immobilization stress [7], and radiation [8]. Leaky gut syndrome (LGS) is an outcome of this inflammatory process. LGS is characterized by impairment in intestinal permeability and the subsequent introduction of undesired pathogens into the body, which might play an important role in multiple diseases [9, 10]. Therefore, if the interaction between LGS and atherosclerosis could be clarified, LGS could provide a potential therapeutic target for atherosclerosis.

Lubiprostone, a ClC-2 chloride channel activator, is a synthetic bicyclic fatty acid derivative of prostablandin E1 that is clinically used as a laxative [11]. Recently, the use of lubiprostone has been shown to have beneficial effects on LGS via stimulation of the intestinal secretion of mucin and trafficking tight junction proteins [12–14]. Therefore, our aim in this study was to investigate the potential effect of lubiprostone on impaired intestinal dysfunction and the development of atherosclerosis in apolipoprotein E-deficient (ApoE-/-) mice.

## Methods

### Animals, diets, and treatment

**Fig 1:**
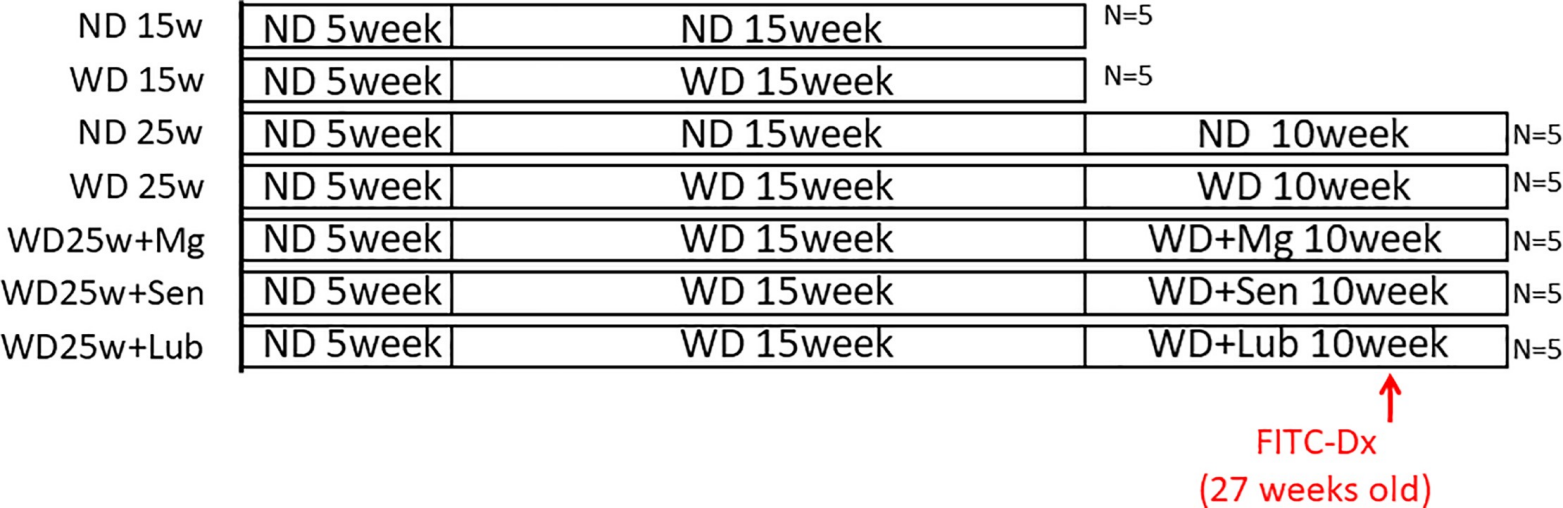
Experimental design. ND; normal diet, WD; western diet, Mg; magnesium hydroxide, Sen; sennoside, Lub; lubiprostone. FITC-Dx; fluorescein isothiocyanate-dextran.

Five-week-old ApoE-/- mice (on the C57BL/6J background) were a generous gift from Dr. Hashimoto (Yokohama City University Graduate School of Medicine, Yokohama, Japan). All mice included in the analysis survived the 30-week duration of the study (5 weeks of adaptation feeding and 25 weeks experimental observation period). Prior to randomized allocation of mice to the experimental and control groups, all mice were provided with a normal diet (ND) and tap water ad libitum for a period of 5 weeks. After this initial period of adaptation, mice were allocated to the following groups. In the ND group (N = 10), mice continued to be fed with the ND for 15 (N = 5) or 25 (N = 5) weeks. In the WD group (N = 25), the ND was switched to a high fat, high cholesterol Western-type diet, containing 21.22% (g/100 g) fat, 17.01% protein, 48.48% carbohydrate, and 0.15% cholesterol; (Oriental Yeast, Tokyo, Japan). After 15 weeks, mice in the WD group were further allocated to the control group (N = 5, continued feeding of the WD for an additional 10 weeks) and the treatment groups, which were administered 500 μg/kg lubiprostone (N = 5) (Sucampo Pharma Americas, Inc.) [15]; 25 mg/kg sennoside (N = 5), an irritant laxative [16]; or 60 mg/kg magnesium hydroxide [Mg(OH)_2_] (N = 5), an osmotic laxative [17] via daily gavage until week 30). Mice, from the ND and WD groups, sacrificed at 20 weeks, provided the baseline measure of the development of atherosclerotic lesions and serum levels of lipid profile. The experimental design is shown in Fig 1.

### In-vivo measurement of intestinal permeability

*In-vivo* permeability of the intestinal wall was quantified as the permeability to 4-kDa fluorescein isothiocyanate (FITC) -dextran (Sigma-Aldrich, St. Louis, MO, USA), as described previously [18]. Briefly, 27-week-old mice (with or without undergoing a 7-week-supplementation of laxatives) underwent oral gavage of FITC-dextran (500 mg/kg) after a fasting period of 6 hours, followed by collection of serum samples via the facial vein. The concentration of FITC-dextran in the serum was measured using fluorescence spectrophotometer (ARVO MX, PerkinElmer, Boston, MA, USA), with an excitation wavelength of 485 nm and an emission wavelength of 535 nm, which allowed a direct assessment of the changes in intestinal barrier function.

### Quantification of atherosclerosis and histologic analyses

After 15 or 25 weeks of consuming the ND or a WD, mice were sacrificed and the heart and aorta of each mouse were harvested for quantification of atherosclerotic lesions using a cross-sectional analysis of the aortic root and en-face analysis of the whole aorta (from the ascending aorta to the common iliac arteries). In detail, mice were anesthetized with isoflurane. After anesthesia was fully effective, mouse limbs were fixed to a cork plate with a pin, and a cut was made in the middle of the abdomen to produce a longitudinal incision from the neck to the lower abdomen. Blood was collected from the right ventricle using 1.0-ml syringes (27G needles) when the heart was completely exposed. After harvesting of the perivascular adipose tissue (PVAT), aortas were fixed in 10% formalin, opened longitudinally, and stained for lipid deposition with Oil Red O solution (Sigma-Aldrich). The area of atherosclerotic lesions was calculated using the ImageJ program (National Institutes of Health, Baltimore, MD, USA). The area of all observed plaques was summed and expressed as a percentage of the total area of the vessel. The hearts were embedded in paraffin and serial 5 μm sections were cut, from the beginning of the 3 aortic leaflets to the ascending aorta, and stained with hematoxylin and eosin (HE). For immunohistochemistry, serial sections were stained with anti-F4/80 antibodies (Abcam, Cambridge, UK), a specific marker for mature macrophages. The intensity in positively stained areas (F4/80-positive areas) was analyzed using ImageJ software. In addition, sections were stained with anti-CD3 antibodies (Abcam), a specific marker for T lymphocytes, and positively stained areas were analyzed.

### Histological analyses of ilea

As well as the hearts, the terminal ilea were embedded in paraffin, and serial 5-μm sections were cut and stained with HE and Masson’s trichrome (MT). Serial sections were then incubated with primary antibodies against two key plexus tight-junctional proteins: ZO-1 (Abcam) and occludin (Abcam).

### Real-time polymerase chain reaction (RT-PCR) analysis

Whole spleen, PVAT, and the ileum were respectively homogenized in TRIzol reagent (Catalog No. 00571510, Thermo Fisher Scientific, Waltham, MA, USA), and total RNA was extracted using a RiboPure Kit (Catalog No. AM1924, Life Technologies, Carlsbad, CA, USA), according to the manufacturer's protocol. A total of 500 ng of RNA was reverse-transcribed to cDNA using a High Capacity RNA-to-cDNA Kit (Catalog No. 4387406, Applied Biosystems, Foster City, CA, USA), according to the manufacturer's protocol. The cDNAs were subsequently mixed with RT2 SYBR Green ROX qPCR Master mix (Catalog No. 1712516, Thermo Fisher Scientific), and RT-PCR was performed in accordance with the manufacturer's instructions. Thermal cycling and fluorescence detection were performed using an ABI7500 RT-PCR machine (Applied Biosystems), according to the manufacturer’s recommendations. The relative mRNA expression levels were determined using glyceraldehyde-3-phosphate dehydrogenase (*Gapdh*) as the housekeeping gene and the 2^−ΔΔCt^ method. The primers used for each gene are listed in S1 Table.

### Quantification of plasma parameters

Whole blood samples were obtained from mice at the time of sacrifice via right ventricular puncture. Low-density lipoprotein cholesterol (LDL-c), high-density lipoprotein cholesterol (HDL-c) and triglyceride (TG) levels were determined by SRL Inc. (SRL Inc., Tokyo, Japan). Total serum IgG was measured in diluted serum using the Mouse IgG ELISA Kit (Bethyl Laboratories, E99-131), according to the manufacturer's instructions. In addition, serum IgG3 was measured using the Mouse IgG3 ELISA Kit (Bethyl Laboratories, E99-111). Data are expressed in micrograms per milliliter (μg/ml), based on the standard curves of isotype standards.

### Statement of ethics

All procedures in this study were conducted in accordance with the animal care guidelines of Yokohama City University Graduate School of Medicine. All of the animal studies were conducted in accordance with the animal care guidelines of Yokohama City University Graduate School of Medicine and approved by IACUC as F-A-17-035.

### Statistical analysis

Continuous data are expressed as means ± standard error of the mean (SEM). One-way ANOVA was used for comparisons among multiple experimental groups and to assess global significance among experimental groups. *A priori* hypothetical differences between groups were evaluated using Student’s *t*-test. Differences with p<0.05 were considered statistically significant. All analyses were performed using JMP 9.1 software (SAS Institute Inc., Cary, NC, USA).

## Results

### Oral supplementation with lubiprostone did not reverse but did attenuate the development of atherosclerosis

**Fig 2:**
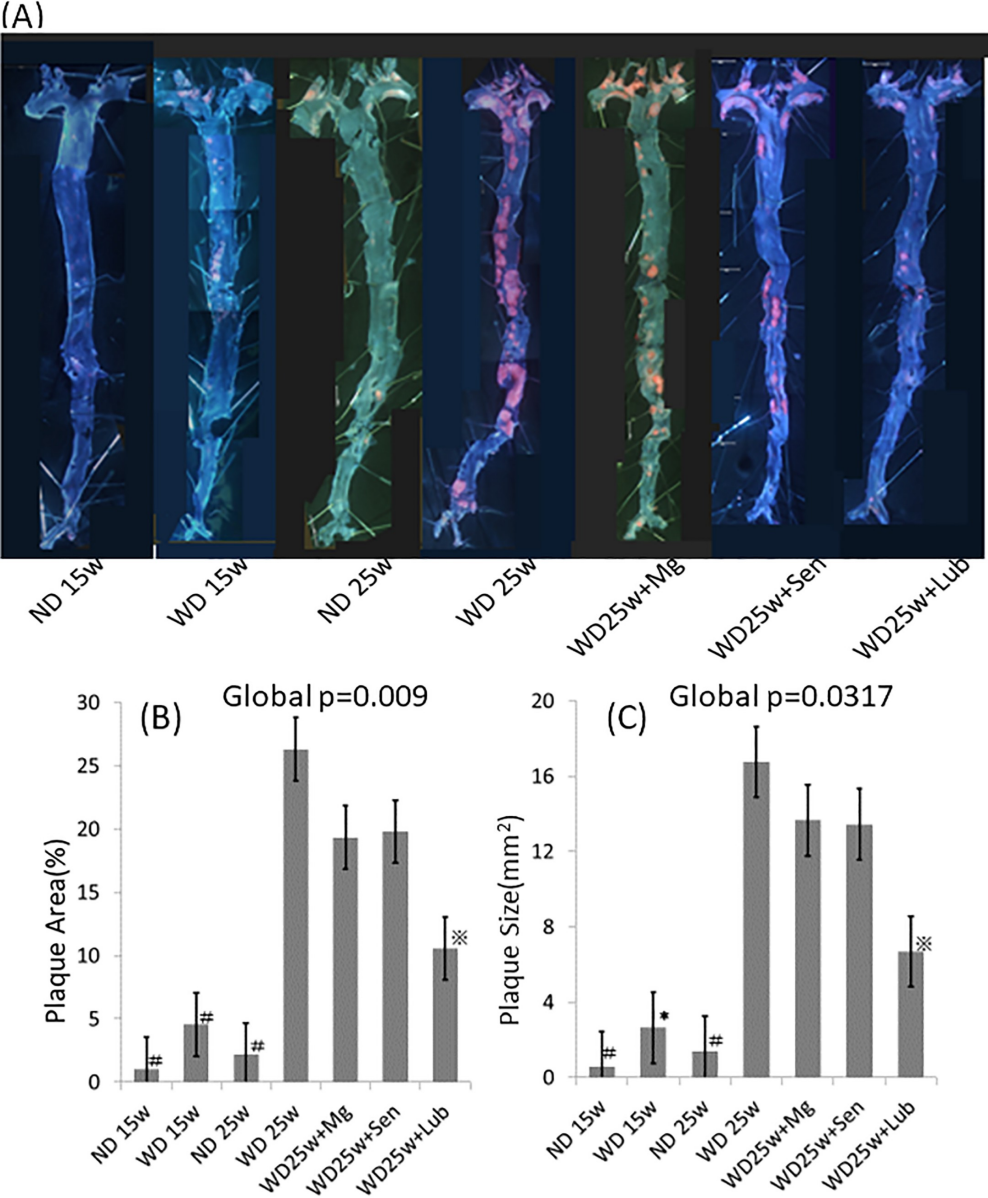
Suppression of the development of atherosclerotic lesions by lubiprostone in longitudinally opened aortas. (A) Representative images of an en-faced aorta, from the aortic arch to the common iliac arteries, visualized using staining Oil Red O. The (B) percentage (%) area of the entire aorta comprising the lesion section and (C) size of atherosclerotic lesions (mm^2^) were analyzed using ImageJ software. Data are presented as the mean±standard error of the mean (SEM), with 5 animals in each group. Global significance among multiple groups was determined by one-way ANOVA. ND; normal diet, WD; western diet, Mg; magnesium hydroxide, Sen; sennoside, Lub; lubiprostone. #:p<0.001, ✽:p = 0.001, ※:p<0.01, compared to the 25-week WD group.

**Fig 3:**
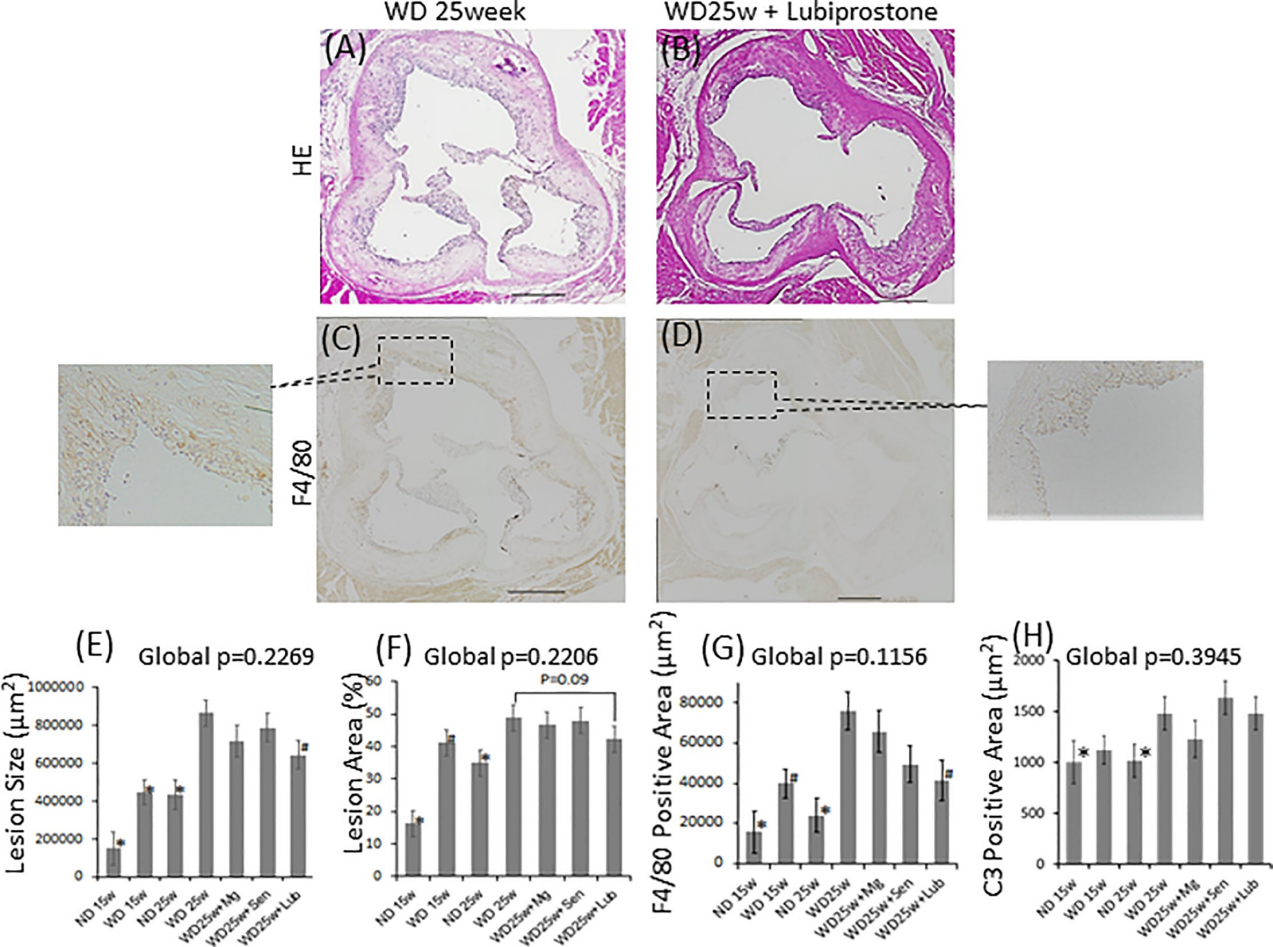
Suppressive effects of lubiprostone on the development of atherosclerotic lesions, quantified on cross-sectional analysis of the aortic root. Representative images of (A) mice in the 25-week WD group and (B) 25-week WD combined with lubiprostone group, analyzed using hematoxylin and eosin staining. The expression of macrophage antigen (F4/80) in each atherosclerotic lesion was detected and visualized by immunohistochemical staining (C and D); higher magnification images are shown for the highlighted areas. Scale bar = 300 μm. (E) Plaque size (μm^2^) and (F) the percentage of plaque-to-vessel area, analyzed using ImageJ software. (G and H) Macrophage infiltration and T cell infiltration were also assessed as the percentage of the F4/80-positive area and CD3-positive area to vessel area, respectively. Data are presented as the mean±standard error of the mean (SEM), with 5 animals in each group. Global significance among multiple groups was determined by one-way ANOVA. HE; hematoxylin and eosin staining. ND; normal diet, WD; western diet, Mg; magnesium hydroxide, Sen; sennoside, Lub; lubiprostone. HE; hematoxylin and eosin staining. ✽:p<0.01, #:p<0.05, compared to the 25-week WD group.

Compared to the ND, a 15-week feeding of the WD induced formation of larger atherosclerotic lesions, as demonstrated by Oil Red O staining of longitudinally opened aortas, which was augmented by an additional 10 weeks of continuous feeding of a WD (Fig 2). The 10-week oral administration of lubiprostone, after the 15-week WD, significantly suppressed the development of atherosclerotic lesions by 69% (p<0.01), compared to that in the 25-week WD group. Histological examination of the aortic roots confirmed a 26% reduction in the area of atherosclerotic lesions (p<0.05, Fig 3). However, of note, in both regions of the longitudinally opened aortas and aortic roots, the atherosclerotic lesion area was still greater in lubiprostone-supplemented mice compared to that at baseline (15-week WD group). These results indicate that oral supplementation with lubiprostone attenuated WD-induced exacerbation of atherosclerotic lesion formation but was not sufficient to induce regression of atherosclerotic plaques. Immunohistochemical analyses of the aortic root demonstrated that lubiprostone significantly reduced macrophage infiltration in plaques, as determined by F4/80 staining (Fig 3C, 3D and 3G; p<0.05). In contrast, T cells infiltrated the plaques only sparsely, and no significant difference was observed between the 25-week WD group and the groups treated with laxative gavage, including lubiprostone (Fig 3H).

### Lubiprostone restored WD-induced intestinal permeability

**Fig 4:**
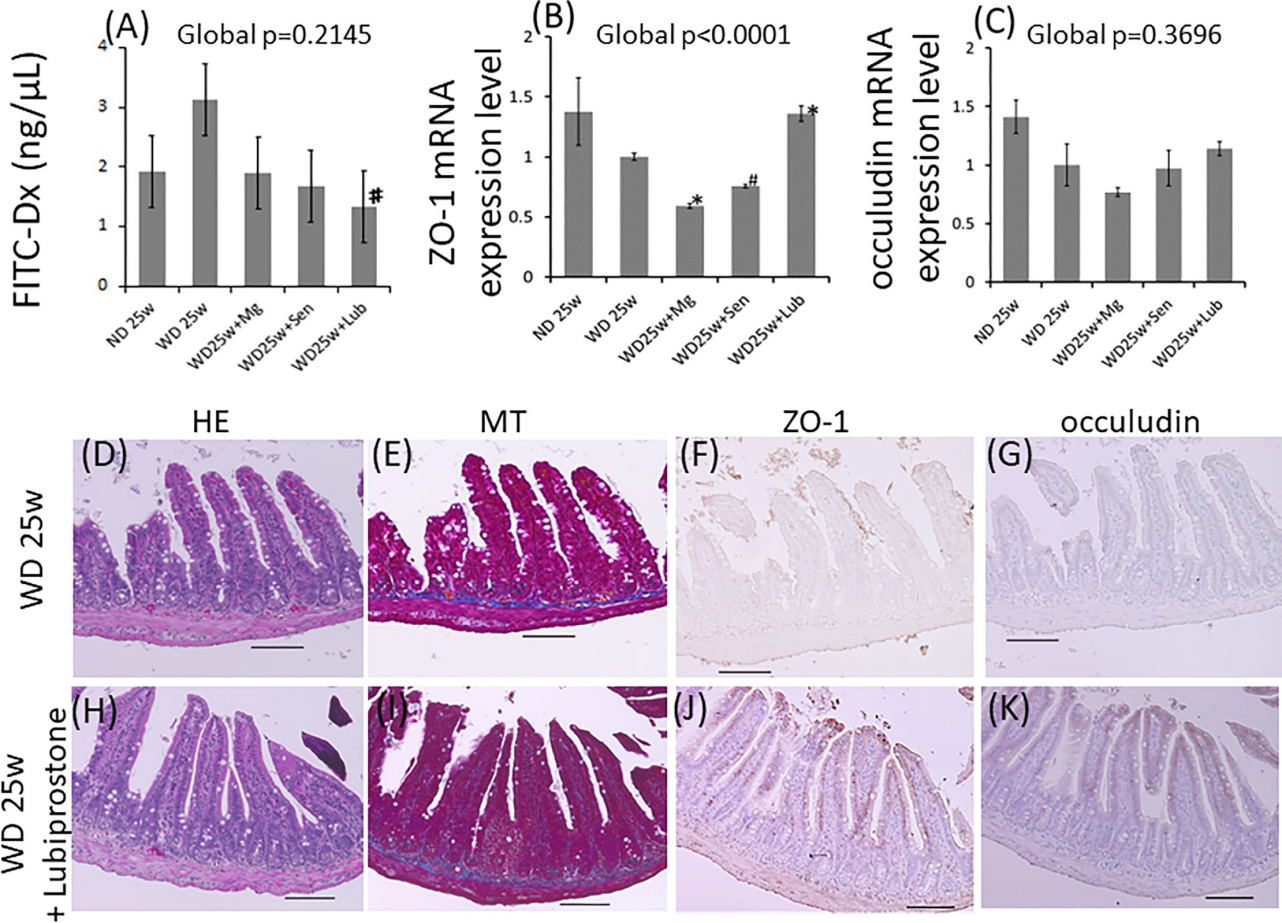
Lubiprostone improved intestinal barrier dysfunction. (A) In vivo intestinal permeability, determined by the measurement of serum concentrations of FITC- dextran, 1 h after oral gavage. (B-C) mRNA levels of ZO-1 and occludin, extracted from the ileum, were determined using qPCR. (D–K) Representative images of the terminal ilea in the 25-week WD group (D and E) and 25-week WD combined with lubiprostone group (H and I), analyzed using hematoxylin and eosin staining and Masson’s trichrome. The expression of tight junction proteins such as ZO-1 (F and J) and Occludin (G and K) in serial sections was detected and visualized by immunohistochemical staining. Scale bar = 100 μm. Global significance among multiple groups was determined by one-way ANOVA. HE; hematoxylin and eosin staining, MT; Masson’s trichrome staining, ND; normal diet, WD; western diet, Mg; magnesium hydroxide, Sen; sennoside, Lub; lubiprostone. ✽:p<0.01, #:p<0.05, compared to the 25-week WD group.

*In vivo* intestinal permeability, as determined by the translocation of orally-administered FITC-dextran to plasma component, was significantly higher in the WD than ND group (Fig 4A). However, in spite of continuous WD-feeding, the appearance of orally administered FITC-dextran was significantly reduced by lubiprostone supplementation. In addition, we investigated the expression of epithelial tight junctions of intestine, which regulate intestinal permeability by blocking the induction of pathogens into the body. The expression of zona occludens protein-1 (ZO-1), an epithelial tight junction protein, was significantly reduced by WD feeding but was up-regulated by supplementation with lubiprostone (Fig 4B). Lubiprostone supplementation also increased the expression of occludin, another tight junction protein (Fig 4C).

We also evaluated histological images of terminal ilea. Although there were no differences in the staining patterns of HE and MT, immunohistochemistry revealed that supplementation with lubiprostone increased the expression of ZO-1 and occludin in villus epithelial cells (Fig 4D–4K). These results suggested that intestinal barrier function improved with oral supplementation of lubiprostone, which might attenuate the release of pathogens into the circulation and prevent LGS-induced systemic inflammation.

### Improvement of intestinal barrier function attenuated WD-induced activation of FOB cells

**Fig 5:**
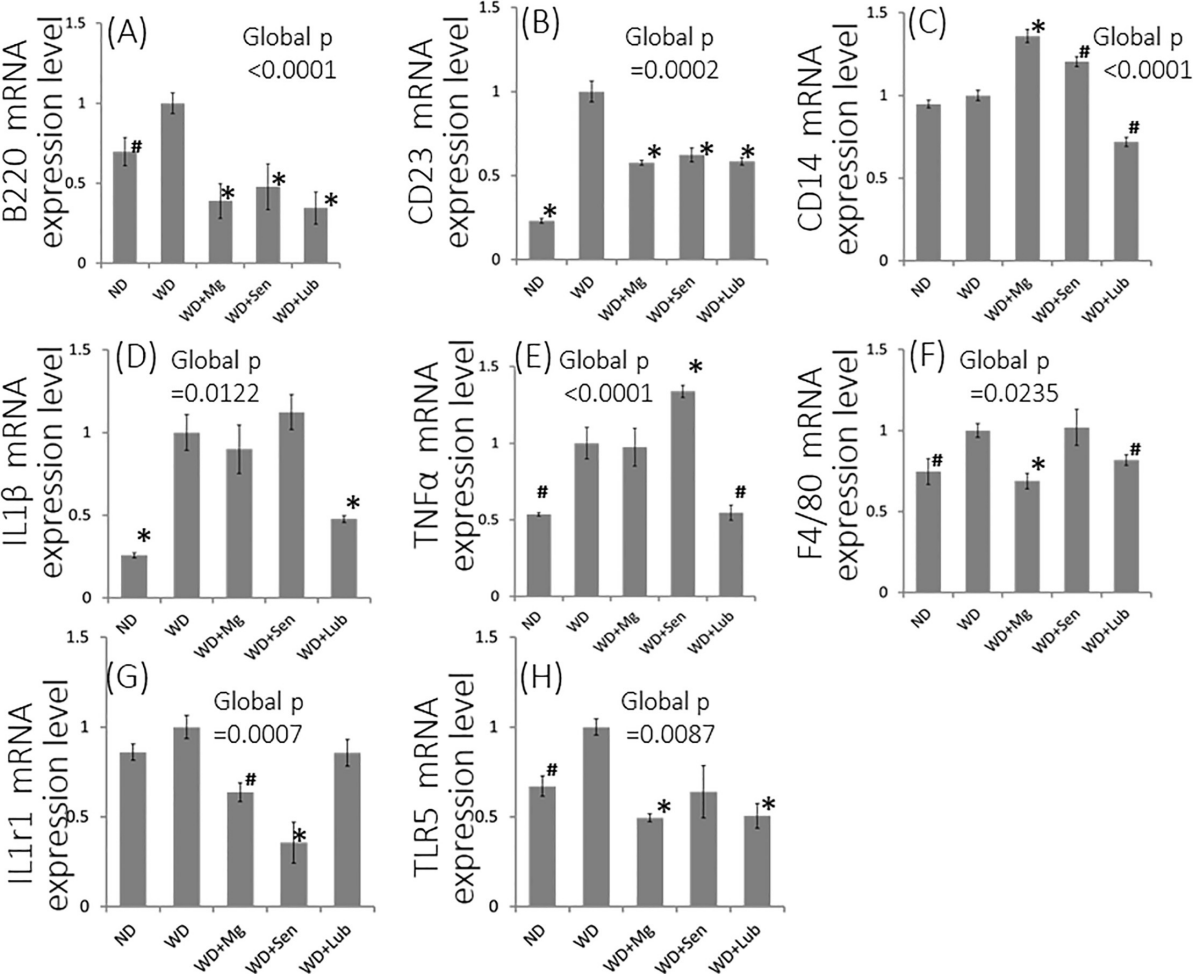
Relative mRNA expression in PVAT was quantified using qPCR and normalized to that of *Gapdh*. The relative ratio is shown compared to mRNA extracted from WD mice. IL1β; interleukin 1 beta, TNFα; tumor necrosis factor-α, IL1r1; interleukin 1 receptor, type 1, TLR5; toll-like receptor 5. Data are presented as the mean±standard error of the mean (SEM), with n = 5 in each group. Global significance among multiple groups was determined by one-way ANOVA. ND; normal diet, WD; western diet, Mg; magnesium hydroxide, Sen; sennoside, Lub; lubiprostone. ✽:p<0.01, #:p<0.05, compared to the 25-week WD group.

Besides the spleen, B cells are also known to reside in PVAT and the adventitial layer of vessels [19, 20]. We previously reported that pronounced B2 cell infiltration was augmented in the PVAT of WD-fed mice, and was an essential contributor to the induction of atherosclerosis [3]. To test the hypothesis that the effects of lubiprostone are exerted via inactivation of B2 cells, we analyzed the aggregation of B2-cells in PVAT. The expression of B220 and CD23, the surface marker of follicular B cell (FOB), was significantly depleted in the mice given a WD with any laxatives compared to those given a WD alone (p<0.001, Fig 5A and 5B). As the most striking difference among the groups with laxatives, the transcription of CD14, which encodes the molecule surface antigen required for B-cell function, was significantly down-regulated in the PVAT of lubiprostone-supplemented mice (Fig 5C). In addition, the genes encoding inflammation-activation related molecules, including interleukin 1 beta (IL1β) and tumor necrosis factor receptor alfa (TNFα), were also significantly down-regulated only in lubiprostone-supplemented mice (Fig 5D and 5E). These findings strongly indicate that the observed effects of lubiprostone supplementation in attenuating the inflammatory phenotype of PVAT and suppressing the development of atherosclerosis were mediated via inactivation of the B2-cell toll-like receptor (TLR) signaling pathway. The expression of F4/80, a surface marker of another inflammatory contributor macrophage, was also reduced in the groups with laxatives (Fig 5F). However, the depletion ratio was not as great as that of FOB surface markers.

**Fig 6:**
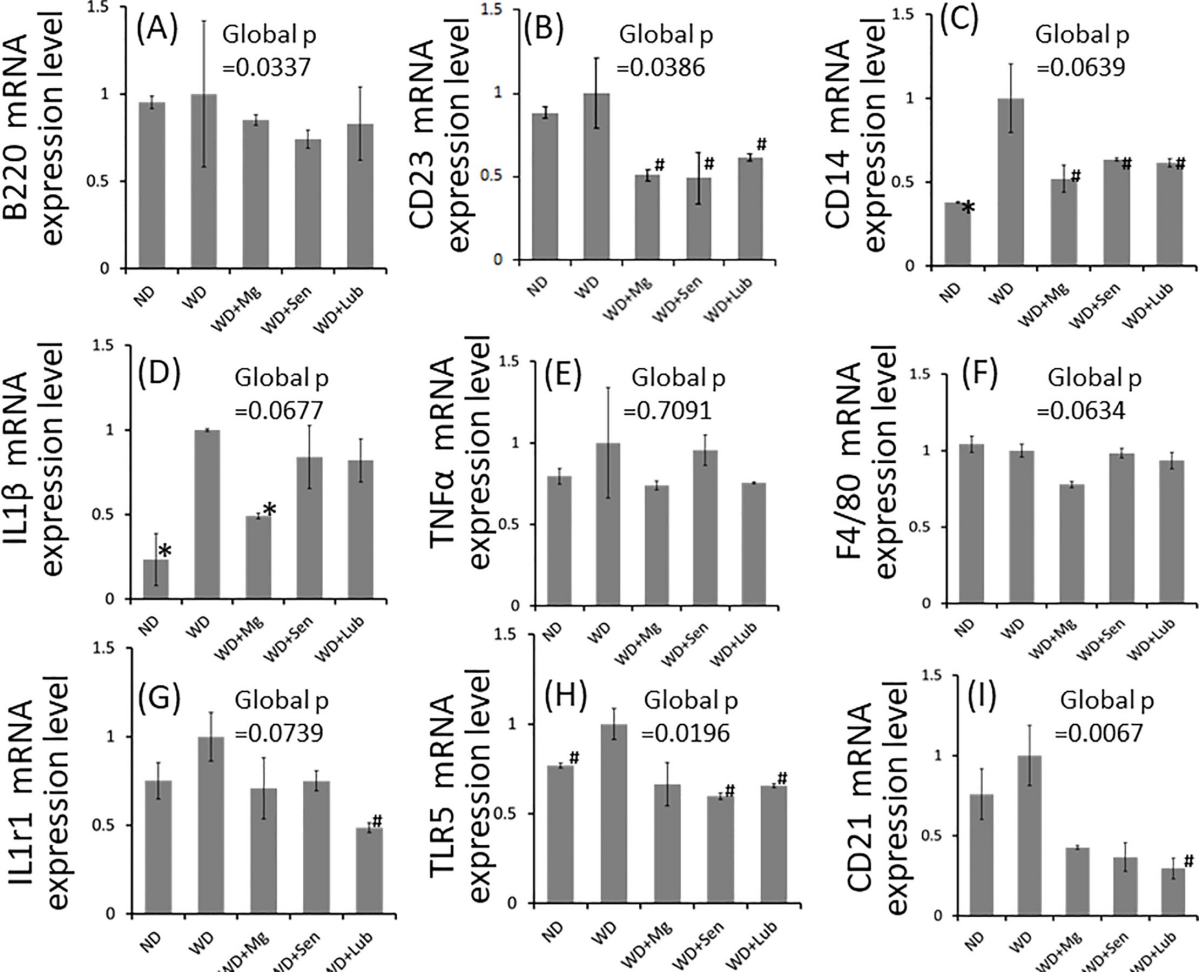
Relative mRNA expression in the spleen, quantified using qPCR and normalized to that of *Gapdh*. The relative ratio is shown compared to the mRNA extracted from WD mice. IL1β; interleukin 1 beta, TNFα; tumor necrosis factor-α, IL1r1; interleukin 1 receptor, type 1, TLR5; toll-like receptor 5. Data are presented as the mean±standard error of the mean (SEM), with n = 5 in each group. Global significance among multiple groups was determined by one-way ANOVA. ND; normal diet, WD; western diet, Mg; magnesium hydroxide, Sen; sennoside, Lub; lubiprostone. ✽:p<0.01, #:p<0.05, compared to the 25-week WD group.

Concomitantly, we also investigated B2 cells in the spleen, the major B cell reservoir [21]. Although there was no significant difference in the expression of B220, CD23 was significantly suppressed in the spleen of laxative-supplemented mice (Fig 6A and 6B). In addition, the expression of CD21, a surface marker of marginal zone B cells (MZB) whose location in the marginal zone of the spleen provides the ability to respond rapidly to blood-borne antigens [22], was suppressed to the greatest extent in lubiprostone-supplemented mice (Fig 6C).

**Fig 7:**
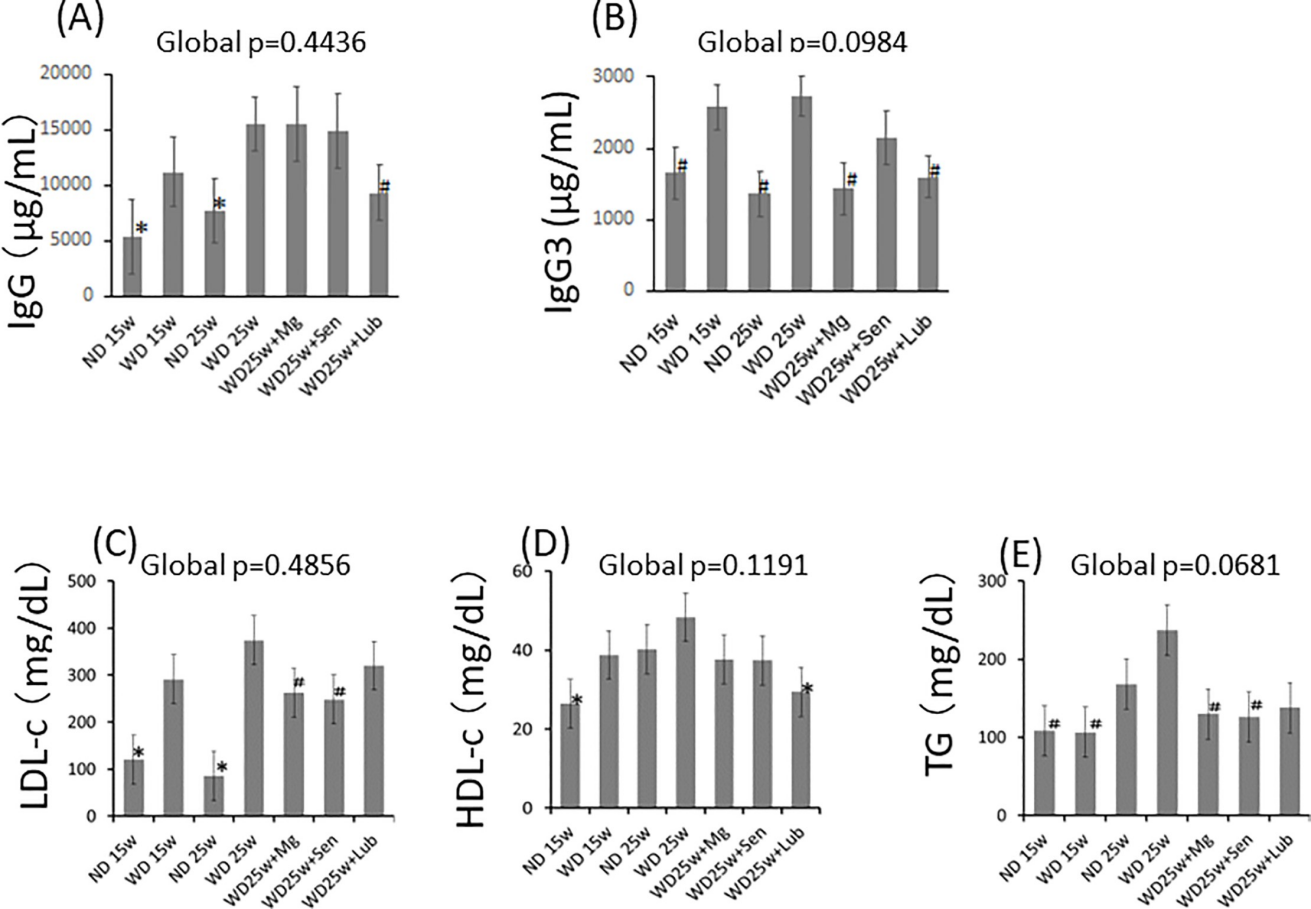
Lubiprostone suppressed the production of immunoglobulin through a lipid-independent pathway. (A and B) Concentration of total IgG and IgG3, assessed using ELISA. (C–E) Serum levels of low-density lipoprotein cholesterol (LDL-c), high-density lipoprotein cholesterol (HDL-c), and triglyceride (TG). Data are presented as the mean±standard error of the mean (SEM). n = 7 for A and B, n = 5 for all other observations. Global significance among multiple groups was determined by one-way ANOVA. ND; normal diet, WD; western diet, Mg; magnesium hydroxide, Sen; sennoside, Lub; lubiprostone. FITC-Dx; fluorescein isothiocyanate-dextran. ✽:p<0.01, #:p<0.05, compared to the 25-week WD group.

The primary function of activated B cells is to produce antibodies. Therefore, we also investigated circulating IgG titers (Fig 7A and 7B). A WD, provided for both 15 and 25 weeks, induced a higher level of IgG, compared to the ND for each duration. In WD-fed mice gavaged with Mg(OH)_2_ and sennoside, the level of IgG was similar to that of WD alone. However, in mice provided with lubiprostone supplementation, total IgG titers were significantly lower than those in mice fed a WD alone (Fig 7A). Moreover, levels of IgG3, a specific subclass of IgG that is mainly produced by MZB cells via a T cell-independent pathway under pre-immune conditions or after immunization [23, 24], were significantly lower in lubiprostone- or Mg(OH)_2_-gavaged mice than in mice in the WD alone group (p<0.05; Fig 7B). These data also suggest that LGS-induced atherosclerosis is characterized by increased antibody production caused by B2-cell activation, which is consistent with data of the RT-PCR analysis.

### Atheroprotective effect of lubiprostone is mediated via a lipid-independent pathway

In Mg(OH)_2_- and sennoside-gavaged mice, serum levels of LDL-c and TG were significantly lower than those in mice in the WD group (Fig 7C and 7D). Meanwhile, in lubiprostone-gavaged mice, there was a slight decrease in serum levels of LDL-c and TG, although this decrease was not significant. However, among the 3 laxatives [Mg(OH)_2_, sennoside, and lubiprostone], only lubiprostone produced a significant decrease in the serum level of HDL-c (Fig 7E), in spite of the superiority of lubiprostone in terms of atheroprotective effects. Collectively, these data suggest that lubiprostone can suppress the progression of atherosclerosis via a lipid metabolism-independent mechanism, although lipid metabolism might endow, at least partially, modest atheroprotective effects in mice with Mg(OH)_2_ or sennoside.

## Discussion

The findings reported in this study demonstrate the potential role of lubiprostone in attenuating atherosclerotic lesions through the amelioration of LGS-induced inflammation by restoring the functional barrier of the gut. WD-induced changes in intestinal barrier function (reduction in intestinal tight junction proteins resulting in increased *in vivo* permeability) led to increased activation of B2 cells and an increment in circulating IgG, accompanied by an up-regulation of pro-inflammatory cytokines and chemokines in the spleen and PVAT. Oral supplementation of lubiprostone attenuated these inflammatory sequelae and suppressed WD-induced exacerbation of atherosclerotic lesion formation, which would be considered to have substantial clinical value.

In the present study, not only were the surface markers of FOB down-regulated in PVAT by lubiprostone supplementation but so was the expression of CD14, which is required for B cell-function. Previous studies have revealed that CD14 is a component of the innate immune system and acts as a co-receptor, along with TLR2 and TLR4, for the detection of bacterial LPS [25–28]. Therefore, the suppression of B2 cell activation, and the subsequent inflammatory response, by lubiprostone-supplementation was likely induced by an improvement in LGS.

With chronic inflammation, tertiary lymphoid organs develop adjacent to diseased tissue, specifically the arterial adventitia and PVAT in the case of atherosclerosis, and may become major sites of adaptive immune activation [19, 28, 29]. It is likely that tertiary lymphoid organs accumulate B cells with relevant antigen specificity [20]. In addition to antigen presentation to CD4 T cell, B cells can potentially aggravate atherosclerosis by at least two other mechanisms, namely the production of atherogenic IgG and the secretion of pro-inflammatory cytokines, including TNFα, which are T cell-independent pathways [29]. Although most inflammatory cells in intimal atherosclerotic plaque are macrophage-derived foam cells and T cells, B cell infiltration was predominant in the adventitial layer of the arterial wall, where tertiary lymphoid organs can be formed [29]. Recently, Tay et. al. reported that B2-derived TNFα promoted atherosclerosis by augmenting TNFα production via lesion macrophages [30]. This increased apoptosis and necrotic cores, via Fas expression, and inflammation of the lesions, via enhanced IL1β and monocyte chemo attractant protein 1 (MCP-1) expression. This indicates that in the atherogenic pathway, B2 cells exist on the upstream side of macrophages and, accordingly, may be key modulators of atherogenesis [30]. This theory might explain why lubiprostone significantly reduced macrophage infiltration in plaques in the aortic roots in our study.

The use of B cell-depleting antibodies, such as anti-CD20 or anti-CD23 antibody, specifically targets B2 cells, reducing their numbers and attenuating their effector [3, 31, 32]. Considering the protective effect of mouse anti-CD20 or 23 treatment in atherosclerotic mice, it would be particularly interesting to investigate the effect of some molecular targeted drugs on cardiovascular risk, such as rituximab, which is widely used for the treatment of patients with rheumatoid arthritis, and lumiliximab, which is not currently marketed due to a failed clinical trial for its use in the treatment of chronic lymphocytic leukemia [33]. However, it is also important to note that long-term B cell depletion could result in an increased risk for infection due to compromised humoral immunity, especially in older patients with cardiovascular risk.

The intestinal microbiota modulates the immune system of the host by producing a wide variety of metabolites or bacterial products [34, 35]. Altered composition and function of intestinal microbiota have been reported to be associated with a number of chronic diseases, including autoimmune disease [36, 37]. Recent reports have suggested that, in the WD-induced exacerbation of atherosclerosis, these changes are considered to be the cause rather than the consequence of pathologies [38, 39]. Therefore, symbiotic or antibiotic therapies, which have been shown to decrease bacterial translocation from the gut in pilot studies [3, 40], might be another therapeutic target for atherosclerosis. However, as well as antibiotic use, attempts to recondition the functional intestinal barrier through the use of so called ‘probiotics’, normally applied to the gut, have had lower success in human than animal studies and are often incompatible with other pharmacological treatments [41, 42]. In addition, the safety and potential harms of these interventions are poorly investigated, especially in compromised or older patients with decreased immunity. This is the reason why another viable therapeutic agent is necessary for improvement of intestinal barrier dysfunction and of the sequential occurrence of atherosclerosis.

Lubiprostone has been shown to provide a protective effect against small intestinal injury in animal models through an increase in prostaglandin E2 via an E-type prostanoid-dependent mechanism which stimulates the intestinal secretion of mucin and trafficking intestinal tight junction proteins [13, 14, 43, 44]. These findings prompted us to examine both light microscopic structures and the expression of tight junction proteins in the ileum end in this study. Both the colon and ileum are major sources of intestinal microbes and function as barriers against microbiome translocation. To evaluate the pathology of LGS for atherosclerosis progression, it is necessary to test not only the ileum but also the colon in terms of intestinal biological barrier function. As only the structure and function of the ileum were evaluated here, this represents a current limitation. In addition, lubiprostone has also been shown to improve disturbances in intestinal permeability, even in humans [45]. Since lubiprostone is generally used as a laxative for chronic idiopathic constipation, its safety is guaranteed, unlike the use of antibiotics and probiotics. As such, lubiprostone would be a candidate as a first line drug to improve LGS-induced atherosclerosis. Our current analyses are limited to the examination of the effects of lubiprostone treatment on leaky gut conditions, with an assumed beneficial change due to improvements in the translocation of the gut microbiome. Previously, dysbiosis of the microbiota has been reported to be responsible for atherosclerosis [39], and lubiprostone treatment has been shown to improve the composition of the microbiota in the dysbiotic state [46]. Therefore, in addition to changes in LGS induced by lubiprostone treatment, changes in the microbiota population may be involved in the anti-atherosclerotic effect of lubiprostone treatment. The strategy suggested here represents a change in the existing clinical paradigm and places the focus on improving intestinal barrier function rather than the direct modulation of gut bacteria itself. Additionally, we did not observe significant changes in body weight (S1 Fig) or stool appearance or weight (S1 and S2 Figs) among the four groups, nor any diarrhea-like symptoms in our experiment (data not shown), suggesting that these changes are not due to nutritional deficiency or dehydration caused by the laxative agents.

In a population-based prospective study of Japanese patients, defecation frequency was found to be inversely related to cardiovascular mortality [47]. Colonic transit time in humans is associated with overall gut microbial mass, composition, diversity, and metabolism, which attenuate not only local but systemic oxidative stress [48, 49]. Additionally, in the present study, laxative treatments themselves might have exerted a modest effect on the microbiota environment by shortening transit time and suppressing the development of atherosclerosis via a decrease in the precursors of toxic metabolic products of the intestinal microbiome. We also showed that laxatives decreased serum lipid levels, which might have influenced the area of plaque formation. However, among the 3 laxatives used, no significant difference was observed in the serum lipid profile, although the severities of LGS and atherosclerosis in lubiprostone-gavaged mice were significantly suppressed compared to those in mice treated with the other 2 laxatives. Therefore aside from its laxative effect, lubiprostone is considered to possess a separate and specific pathway for athero-protection via restoration of the injured intestinal barrier.

In conclusion, lubiprostone attenuates the development of atherosclerotic lesions by ameliorating LGS-induced inflammation via the restoration of the intestinal barrier. These findings raise the possibility of targeting intestinal barrier dysfunction for the treatment of atherosclerosis, rather than direct modulation of intestinal microbiota itself.

## Supporting information

S1 Fig Average body weight and stool weight at the time of sacrifice. Data are shown as the mean±standard error of the mean (SEM), with 5 animals in each group. Global significance among multiple groups was determined by one-way ANOVA.
(TIF)

S2 Fig Representative appearance of stool in each group. (TIF)

S1 Table Sequences of primers used in this study. (DOCX)

## References

[1] Hansson GK. Atherosclerosis—an immune disease: The Anitschkov Lecture 2007. Atherosclerosis. 2009;202: 2–10. 10.1016/j.atherosclerosis.2008.08.039 18951547

[2] Libby P. Inflammation in atherosclerosis. Nature. 2002;420: 868–874. Review. 10.1038/nature01323 12490960

[3] Chen L, Ishigami T, Nakashima-Sasaki R, Kino T, Doi H, Minegishi S, et al Commensal microbe-specific activation of B2 cell subsets contributes to atherosclerosis development independently of lipid metabolism. EBioMedicine. 2016;13: 237–247. 10.1016/j.ebiom.2016.10.030 27810309 PMC5264349

[4] Ishigami T, Abe K, Aoki I, Minegishi S, Ryo A, Matsunaga S, et al Anti-interleukin-5 and multiple autoantibodies are associated with human atherosclerotic diseases and serum interleukin-5 levels. FASEB J. 2013;27: 3437–3445. 10.1096/fj.12-222653 23699176

[5] Keita AV, Söderholm JD. The intestinal barrier and its regulation by neuroimmune factors. Neurogastroenterol Motil. 2010;22: 718–733. 10.1111/j.1365-2982.2010.01498.x 20377785

[6] Enomoto N, Ikejima K, Yamashina S, Hirose M, Shimizu H, Kitamura T, et al Kupffer cell sensitization by alcohol involves increased permeability to gut-derived endotoxin. Alcohol Clin Exp Res. 2001;25: 51S–54S. 11410742 10.1097/00000374-200106001-00012

[7] Rivera CA, Bradford BU, Seabra V, Thurman RG. Role of endotoxin in the hypermetabolic state after acute ethanol exposure. Am J Physiol. 1998;275: G1252–G1258. 10.1152/ajpgi.1998.275.6.G1252 9843760

[8] Paulos CM, Wrzesinski C, Kaiser A, Hinrichs CS, Chieppa M, Cassard L, et al Microbial translocation augments the function of adoptively transferred self/tumor-specific CD8+ T cells via TLR4 signaling. J Clin Invest. 2007;117: 2197–2204. 10.1172/JCI32205 17657310 PMC1924500

[9] Perrier C, Corthésy B. Gut permeability and food allergies. Clin Exp Allergy. 2011;41: 20–28. 10.1111/j.1365-2222.2010.03639.x 21070397

[10] Fresko I, Hamuryudan V, Demir M, Hizli N, Sayman H, Melikoğlu M, et al Intestinal permeability in Behçet's syndrome. Ann Rheum Dis. 2001;60: 65–66. 10.1136/ard.60.1.65 11114285 PMC1753363

[11] Lacy BE, Levy LC. Lubiprostone: a novel treatment for chronic constipation. Clin Interv Aging. 2008;3: 357–364. 18686757 10.2147/cia.s2938 PMC2546479

[12] Moeser AJ, Nighot PK, Roerig B, Ueno R, Blikslager AT. Comparison of the chloride channel activator lubiprostone and the oral laxative Polyethylene Glycol 3350 on mucosal barrier repair in ischemic-injured porcine intestine. World J Gastroenterol. 2008;14: 6012–6017. 10.3748/wjg.14.6012 18932279 PMC2760184

[13] Nighot PK, Blikslager AT. Chloride channel ClC-2 modulates tight junction barrier function via intracellular trafficking of occludin. Am J Physiol Cell Physiol. 2012;302: C178–C187. 10.1152/ajpcell.00072.2011 21956164

[14] De Lisle RC. Lubiprostone stimulates small intestinal mucin release. BMC Gastroenterol. 2012;12: 156 10.1186/1471-230X-12-156 23130661 PMC3523065

[15] Sucampo Pharma Americas, Inc. Amitiza (lubiprostone) capsules label. Available from: https://www.accessdata.fda.gov/drugsatfda_docs/label/2012/021908s010lbl.pdf

[16] Purdue Pharma. Senokot®·S prescribing information. Available from: https://pdf.hres.ca/dpd_pm/00038721.pdf

[17] Pharmaceutical Associates, Inc. MILK OF MAGNESIA- magnesium hydroxide suspension. Available from: https://dailymed.nlm.nih.gov/dailymed/fda/fdaDrugXsl.cfm?setid=e7874dc4-0389-404e-a443-f3d00b2e6528&type=display

[18] Wang Q, Fang CH, Hasselgren PO. Intestinal permeability is reduced and IL-10 levels are increased in septic IL-6 knockout mice. Am J Physiol Regul Integr Comp Physiol. 2001;281: R1013–R1023. 10.1152/ajpregu.2001.281.3.R1013 11507020

[19] Gräbner R, Lötzer K, Döpping S, Hildner M, Radke D, Beer M, et al Lymphotoxin beta receptor signaling promotes tertiary lymphoid organogenesis in the aorta adventitia of aged ApoE-/- mice. J Exp Med. 2009;206: 233–248. 10.1084/jem.20080752 19139167 PMC2626665

[20] Hamze M, Desmetz C, Berthe ML, Roger P, Boulle N, Brancherau P, et al Characterization of resident B cells of vascular walls in human atherosclerotic patients. J Immunol. 2013;191: 3006–3016. 10.4049/jimmunol.1202870 23956434

[21] Tsiantoulas D, Sage AP, Mallat Z, Binder CJ. Targeting B cells in atherosclerosis: closing the gap from bench to bedside. Arterioscler Thromb Vasc Biol. 2015;35: 296–302. 10.1161/ATVBAHA.114.303569 25359862

[22] Cerutti A, Cols M, Puga I. Marginal zone B cells: virtues of innate-like antibody-producing lymphocytes. Nat Rev Immunol. 2013;13: 118–132. 10.1038/nri3383 23348416 PMC3652659

[23] Guinamard R, Okigaki M, Schlessinger J, Ravetch JV. Absence of marginal zone B cells in Pyk-2-deficient mice defines their role in the humoral response. Nat Immunol. 2000;1: 31–36. 10.1038/76882 10881171

[24] Panda S, Zhang J, Tan NS, Ho B, Ding JL. Natural IgG antibodies provide innate protection against ficolin-opsonized bacteria. EMBO J. 2013;32: 2905–2919. 10.1038/emboj.2013.199 24002211 PMC3831310

[25] Tasaka S, Ishizaka A, Yamada W, Shimizu M, Koh H, Hasegawa N, et al Effect of CD14 blockade on endotoxin-induced acute lung injury in mice. Am J Respir Cell Mol Biol. 2003;29: 252–258. 10.1165/rcmb.2002-0132OC 12639839

[26] Heumann D, Adachi Y, Le Roy D, Ohno N, Yadomae T, Glauser MP, et al Role of plasma, lipopolysaccharide-binding protein, and CD14 in response of mouse peritoneal exudate macrophages to endotoxin. Infect Immun. 2001;69: 378–385. 10.1128/IAI.69.1.378-385.2001 11119527 PMC97893

[27] Houtkamp MA, de Boer OJ, van der Loos CM, van der Wal AC, Becker AE. Adventitial infiltrates associated with advanced atherosclerotic plaques: structural organization suggests generation of local humoral immune responses. J Pathol. 2001;193: 263–269. 10.1002/1096-9896(2000)9999:9999<::AID-PATH774>3.0.CO;2-N 11180175

[28] Kyaw T, Tipping P, Bobik A, Toh BH. Opposing roles of B lymphocyte subsets in atherosclerosis. Autoimmunity. 2017;50: 52–56. 10.1080/08916934.2017.1280669 28166680

[29] Mohanta SK, Yin C, Peng L, Srikakulapu P, Bontha V, Hu D, et al Artery tertiary lymphoid organs contribute to innate and adaptive immune responses in advanced mouse atherosclerosis. Circ Res. 2014;114: 1772–1787. 10.1161/CIRCRESAHA.114.301137 24855201

[30] Tay C, Liu YH, Hosseini H, Kanellakis P, Cao A, Peter K, et al B-cell-specific depletion of tumour necrosis factor alpha inhibits atherosclerosis development and plaque vulnerability to rupture by reducing cell death and inflammation. Cardiovasc Res. 2016;111: 385–397. 10.1093/cvr/cvw186 27492217

[31] Kyaw T, Tay C, Khan A, Dumouchel V, Cao A, To K, et al Conventional B2 B cell depletion ameliorates whereas its adoptive transfer aggravates atherosclerosis. J Immunol. 2010;185: 4410–4419. 10.4049/jimmunol.1000033 20817865

[32] Ait-Oufella H, Herbin O, Bouaziz JD, Binder CJ, Uyttenhove C, Laurans L, et al B cell depletion reduces the development of atherosclerosis in mice. J Exp Med. 2010;207: 1579–1587. 10.1084/jem.20100155 20603314 PMC2916123

[33] Awan FT, Hillmen P, Hellmann A, Robak T, Hughes SG, Trone D, et al A randomized, open-label, multicentre, phase 2/3 study to evaluate the safety and efficacy of lumiliximab in combination with fludarabine, cyclophosphamide and rituximab versus fludarabine, cyclophosphamide and rituximab alone in subjects with relapsed chronic lymphocytic leukaemia. Br J Haematol. 2014;167: 466–477. 10.1111/bjh.13061 25130401

[34] Gao Z, Yin J, Zhang J, Ward RE, Martin RJ, Lefevre M, et al Butyrate improves insulin sensitivity and increases energy expenditure in mice. Diabetes. 2009;58: 1509–1517. 10.2337/db08-1637 19366864 PMC2699871

[35] Cani PD, Amar J, Iglesias MA, Poggi M, Knauf C, Bastelica D, et al Metabolic endotoxemia initiates obesity and insulin resistance. Diabetes. 2007;56: 1761–1772. 10.2337/db06-1491 17456850

[36] de Goffau MC, Luopajärvi K, Knip M, Ilonen J, Ruohtula T, Härkönen T, et al Fecal microbiota composition differs between children with β-cell autoimmunity and those without. Diabetes. 2013;62: 1238–1244. 10.2337/db12-0526 23274889 PMC3609581

[37] Manichanh C, Rigottier-Gois L, Bonnaud E, Gloux K, Pelletier E, Frangeul L, et al Reduced diversity of faecal microbiota in Crohn’s disease revealed by a metagenomic approach. Gut. 2006;55: 205–211. 10.1136/gut.2005.073817 16188921 PMC1856500

[38] Li J, Lin S, Vanhoutte PM, Woo CW, Xu A. *Akkermansia muciniphila* protects against atherosclerosis by preventing metabolic endotoxemia-induced inflammation in Apoe-/- mice. Circulation. 2016;133: 2434–2446. 10.1161/CIRCULATIONAHA.115.019645 27143680

[39] Yoshida N, Emoto T, Yamashita T, Watanabe H, Hayashi T, Tabata T, et al *Bacteroides vulgatus* and *Bacteroides dorei* reduce gut microbial lipopolysaccharide production and inhibit atherosclerosis. Circulation. 2018;138: 2486–2498. 10.1161/CIRCULATIONAHA.118.033714 30571343

[40] Bengmark S. Gut microbiota, immune development and function. Pharmacol Res. 2013;69: 87–113. 10.1016/j.phrs.2012.09.002 22989504

[41] Bafeta A, Koh M, Riveros C, Ravaud P. Harms reporting in randomized controlled trials of interventions aimed at modifying microbiota: A systematic review. Ann Intern Med. 2018;169: 240–247. 10.7326/M18-0343 30014150

[42] Andraws R, Berger JS, Brown DL. Effects of antibiotic therapy on outcomes of patients with coronary artery disease: a meta-analysis of randomized controlled trials. JAMA. 2005;293: 2641–2647. 10.1001/jama.293.21.2641 15928286

[43] Hayashi S, Kurata N, Yamaguchi A, Amagase K, Takeuchi K. Lubiprostone prevents nonsteroidal anti-inflammatory drug-induced small intestinal damage by suppressing the expression of inflammatory mediators via EP4 receptors. J Pharmacol Exp Ther. 2014;349: 470–479. 10.1124/jpet.114.213991 24713141

[44] Bassil AK, Borman RA, Jarvie EM, McArthur-Wilson RJ, Thangiah R, Sung EZ, et al Activation of prostaglandin EP receptors by lubiprostone in rat and human stomach and colon. Br J Pharmacol. 2008;154: 126–135. 10.1038/bjp.2008.84 18332851 PMC2438971

[45] Kato T, Honda Y, Kurita Y, Iwasaki A, Sato T, Kessoku T, et al Lubiprostone improves intestinal permeability in humans, a novel therapy for the leaky gut: A prospective randomized pilot study in healthy volunteers. PLoS One. 2017;12: e0175626 10.1371/journal.pone.0175626 28410406 PMC5391961

[46] Mishima E, Fukuda S, Shima H, Hirayama A, Akiyama Y, Takeuchi Y, et al Alteration of the intestinal environment by lubiprostone is associated with amelioration of adenine-induced CKD. J Am Soc Nephrol. 2015;26: 1787–1794. 10.1681/ASN.2014060530 25525179 PMC4520171

[47] Honkura K, Tomata Y, Sugiyama K, Kaiho Y, Watanabe T, Zhang S, et al Defecation frequency and cardiovascular disease mortality in Japan: The Ohsaki cohort study. Atherosclerosis. 2016;246: 251–256. 10.1016/j.atherosclerosis.2016.01.007 26812003

[48] Roager HM, Hansen LB, Bahl MI, Frandsen HL, Carvalho V, Gøbel RJ, et al Colonic transit time is related to bacterial metabolism and mucosal turnover in the gut. Nat Microbiol. 2016;1: 16093 10.1038/nmicrobiol.2016.93 27562254

[49] Stephen AM, Wiggins HS, Cummings JH. Effect of changing transit time on colonic microbial metabolism in man. Gut. 1987;28: 601–609. 10.1136/gut.28.5.601 3596341 PMC1432874

