# Supplementary figures and images for: Lubiprostone as a potential therapeutic agent to improve intestinal permeability and prevent the development of atherosclerosis in apolipoprotein E-deficient mice
Source: PLoS One. 2019 Jun 17;14(6):e0218096. doi: 10.1371/journal.pone.0218096 (PMC6576757; doi:10.1371/journal.pone.0218096)

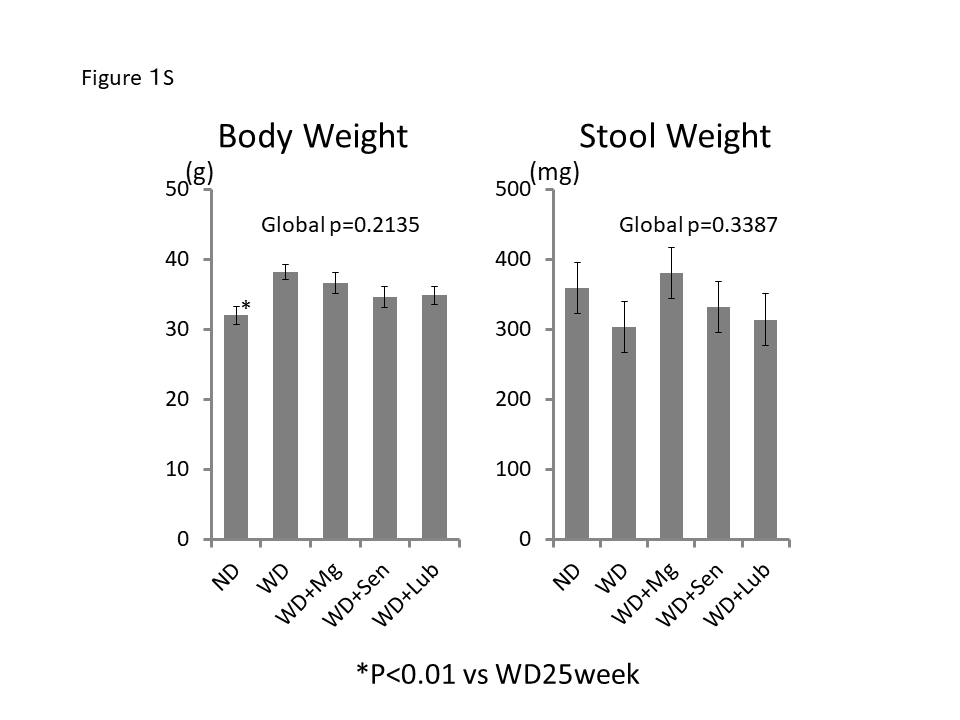

Supplement: S1 Fig — Data are shown as the mean±standard error of the mean (SEM), with 5 animals in each group. Global significance among multiple groups was determined by one-way ANOVA. (TIF) [file pone.0218096.s001.TIF]

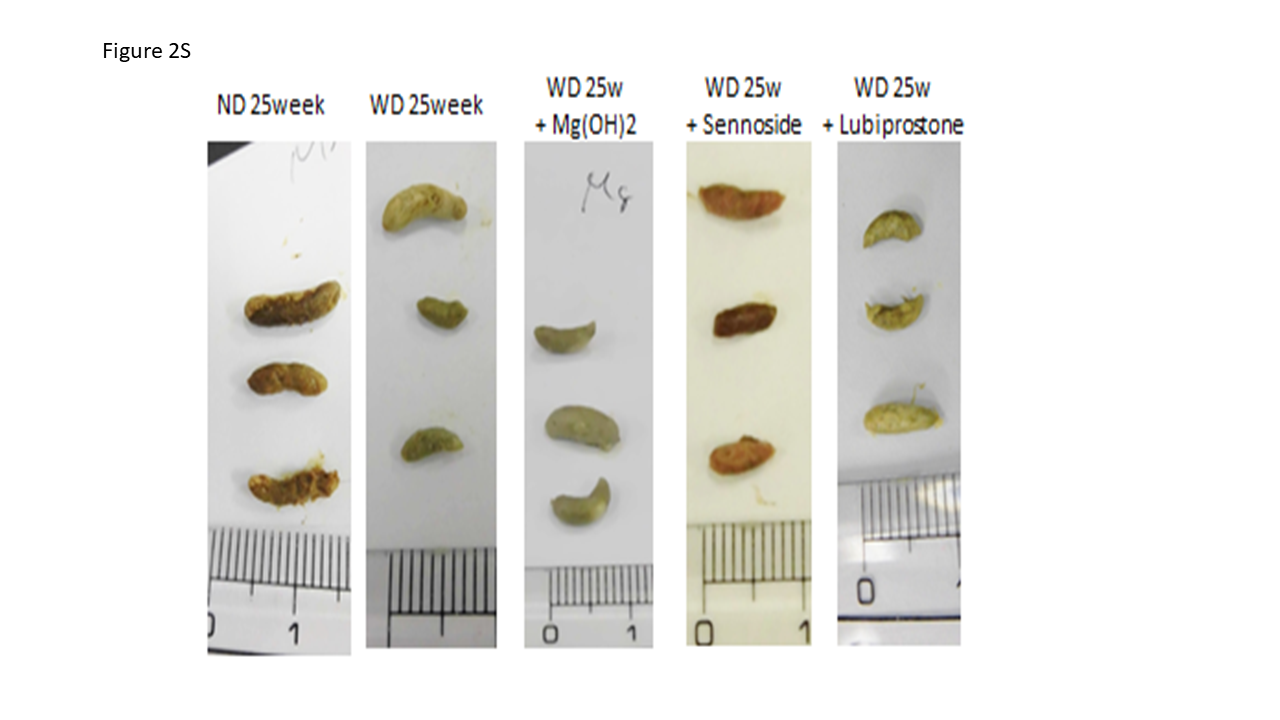

Supplement: S2 Fig — (TIF) [file pone.0218096.s002.TIF]
