# Supplementary material for: Lubiprostone as a potential therapeutic agent to improve intestinal permeability and prevent the development of atherosclerosis in apolipoprotein E-deficient mice
Source: PLoS One. 2019 Jun 17;14(6):e0218096. doi: 10.1371/journal.pone.0218096 (PMC6576757; doi:10.1371/journal.pone.0218096)
Supplement: S1 Table — (DOCX) [file pone.0218096.s003.docx]

**S1 Table. Sequences of primers used in this study**

| **Gene** | **Primer Sequence** |
| --- | --- |
| GAPDH (mouse) | Forward: TGTGAACGGATTTGGCCGTA  Reverse: ACTGTGCCGTTGAATTTGCC |
| ZO-1 (mouse) | Forward: TTTTTGACAGGGGGAGTGG  Reverse: TGCTGCAGAGGTCAAAGTTCAAG |
| Occludin (mouse) | Forward: ATGTCCGGCCGATGCTCTC  Reverse: TTTGGCTGCTCTTGGGTCTGTAT |
| B220 (mouse) | Forward: CACATATCATCCAGGTGTGTTATCC  Reverse: GTCCTCTCCCTGGCACACCTG |
| CD23 (mouse) | Forward: CTCTCCCAGAACCTGAACAGACTC  Reverse: AGCCCTTGCCAAAATAGTAGCAC |
| CD14 (mouse) | Forward: CTGATCTCAGCCCTCTGTCC  Reverse: GTCCCAGCCCAGTGAAAGAC |
| IL1β (mouse) | Forward: GCCCATCCTCTGTGACTCAT  Reverse: AGGCCACAGGTATTTTGTCG |
| TNFα (mouse) | Forward: ACGGCATGGATCTCAAAGAC  Reverse: AGATAGCAAATCGGCTGACG |
| F4/80 (mouse) | Forward: CTTTGGCTATGGGCTTCCAGTC  Reverse: GCAAGGAGGACAGAGTTTATCGTG |
| IL1r1 (mouse) | Forward: GTGCTACTGGGGCTCATTTGT  Reverse: GGAGTAAGAGGACACTTGCGAAT |
| TLR5 (mouse) | Forward: AAGACTGCGATGAAGAGGAAGCCA  Reverse: TGTCCTTGAACACCAGCTTCTGGA |
| CD21 (mouse) | Forward: ATGGGATCCTTGGGTTCGCTC  Reverse: GCTAGGTGAACAAGTGTACCT |
